# Design of an efficient combined multipoint picking scheme for tea buds

**DOI:** 10.3389/fpls.2022.1042035

**Published:** 2022-11-22

**Authors:** Lijia Xu, Yi Xie, Xinyuan Chen, Yanjun Chen, Zhiliang Kang, Peng Huang, Zhiyong Zou, Yong He, Ning Yang, Yingqi Peng, Jianwu Dai, Zhijun Wu, Bi Liu, Yuchao Wang, Yongpeng Zhao

**Affiliations:** ^1^College of Mechanical and Electrical Engineering, Sichuan Agriculture University, Ya’an, China; ^2^College of Engineering, Chinese University of Hong Kong, Hong Kong, Hong Kong SAR, China; ^3^School of Electrical and Information Engineering, Jiangsu University, Zhenjiang, China; ^4^College of Biosystems Engineering and Food Science, Zhejiang University, Hangzhou, China

**Keywords:** tea buds, picking box sets, greedy algorithm, Graham algorithm, minimum bounding box

## Abstract

Herein, a combined multipoint picking scheme was proposed, and the sizes of the end of the bud picker were selectively designed. Firstly, the end of the bud picker was abstracted as a fixed-size picking box, and it was assumed that the tea buds in the picking box have a certain probability of being picked. Then, the picking box coverage and the greedy algorithm were designed to make as few numbers of picking box set as possible to cover all buds to reduce the numbers of picking. Furthermore, the Graham algorithm and the minimum bounding box were applied to fine-tune the footholds of each picking box in the optimal coverage picking box set, so that the buds were concentrated in the middle of the picking boxes as much as possible. Moreover, the geometric center of each picking box was taken as a picking point, and the ant colony algorithm was used to optimize the picking path of the end of the bud picker. Finally, by analyzing the influence of several parameters on the picking performance of the end of the bud picker, the optimal sizes of the picking box were calculated successfully under different conditions. The experimental results showed that the average picking numbers of the combined multipoint picking scheme were reduced by 31.44%, the shortest picking path was decreased by 11.10%, and the average consumed time was reduced by 50.92% compared to the single-point picking scheme. We believe that the proposed scheme can provide key technical support for the subsequent design of intelligent bud-picking robots.

## Introduction

China is a major tea producer, ranking first place in the world with an annual tea production of over 1 Mt. There are many problems in the tea picker, such as high labor intensity, low efficiency, and so on. In particular, the buds of tea are mainly picked manually. According to the survey, since the growth cycle of spring buds is only about 1 week, the best picking time will be missed if they are not picked on time. At the same time, labor costs account for about 50% of tea farmers’ income. Therefore, the intelligent bud picker has become a core technology to be urgently solved (Han et al., 2014). Currently, the machine vision technology has been applied widely in agriculture picking (Chen et al., 2020; Tang et al., 2020; Chen et al., 2021). Many tea-picking machines use this technology to identify and locate multiple buds and then pick them one by one. Obviously, the picking efficiency of this picking method is very low, and the requirements for bud positioning are also quite high. Optimizing the picking path is the key point to improve picking efficiency, and a better picking plan is designed under the premise of ensuring the integrity of the tea buds as much as possible. In 1910, Japan developed single-handed, double-lift, self-propelled tea pickers (Du et al., 2018). In 1959, China included tea pickers as one of the key national research directions. Since then, many tea-picking machines have been developed one after another, but the intelligent picking robot is still in the development stage and has not yet been put into practical application. For example, the No. 4CZ-12 tea picker (Qin et al., 2014) developed by Nanjing agricultural machinery research institute of China can adaptively adjust the height, width, and center of gravity of the bud plane and has a precise bud positioning system based on the machine vision. However, it failed to promote the application because of low work efficiency. Tang et al. (2016) proposed a razor-type tea picker based on the machine vision. The cutting knife is automatically leveled and heightened to make the cutting table consistent with the horizontal plane, but it is difficult to ensure the integrity of the buds. Lu et al. (2015) designed a two-arm linkage synchronous tea-picking machine, which has high picking efficiency. However, due to the short two-axis mechanical arm, it is inconvenient to cover a wider picking area. Yuan et al. (2017) designed a brand tea-picking robot in a rectangular coordinate system. According to the principle of equal number of buds, the picking area was allocated from the left and right rectangles to the two manipulators of the picking robot, and an M-type picking method with the shortest picking path was proposed. In addition, the end of the bud picker can only pick one bud at a time, which leads to a long time for high-density bud picking. The abovementioned picking methods ignore the size of the end of the picker and the high-density buds. Thus, a combined multipoint picking scheme is designed for the densely gathered buds. The experimental results are compared and analyzed, and the optimal sizes of the end of the bud picker are selected to achieve the best picking performance.

The rest of the paper is organized as follows: first, the working principle of the end bud picker, and then the mathematical model of the end bud picker are constructed. After that the busbar pair and solute of the MWC with the broadest coverage of the bud picking box are constructed, and the picking performance of the two picking schemes is compared, the experimental results are analyzed. Finally, summarizes the characteristics of the combined multi-point pickup scheme introduced in this study and provides a conclusion.

### Working principle of the end of the bud picker

**Figure 1:**
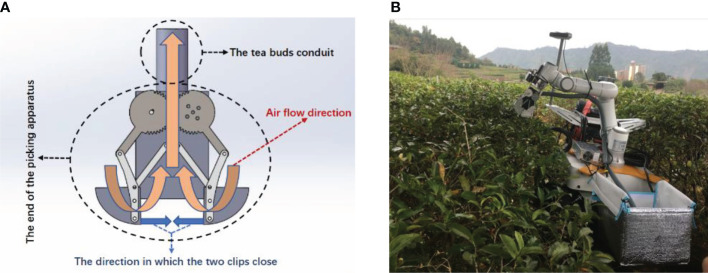
The picking robot and the end of the bud picker. **(A)** Structural diagram of the end of the bud picker. **(B)** The picking robot in the field.

The bud picker is composed of a charge-coupled device (CCD) camera (Wang C. et al., 2017), manipulators with the end of the bud pickers installed on theirs tops, a microprocessor module, a collection box, and an upper computer, which are all mounted on the mobile platform. The CCD camera takes the pictures of the tea ridge to the host computer that obtains the spatial coordinates and distribution density of the buds in the picking area through the machine vision and image processing technology (Zhang et al., 2014; Li et al., 2020; Chen et al., 2020). The host computer plans the picking path at the end of the picking machine and transmits it to the microprocessor module. The microprocessor module sends the commands to the driving device to drive the manipulator to pick (Wang H. et al., 2018). The picked buds are sucked into the collection box through the conduit. The manipulators are driven by the stepper motor, and they move in four directions of *X*, *Y*, *Z*, and *θ*. *XYZ* comprise the spatial coordinate system (Chen et al., 2020), and *θ* is the rotation angle of the manipulator in the *XOY* plane that is the included angle between the length of the end of the bud picker and the *X* axis. The end of the bud picker comprises a pair of meshing driving-driven gears, a pair of elongated clamping fingers, and a conduit. The clamping fingers are connected to the gears by hinges accordingly. The stepping motor drives the gears to make the clamping fingers move and pick the buds by clamping and lifting. The clamping and lifting experiment has been completed in advance to determine the clamping force of the two fingers. The structure of the end of the bud picker and the picking robot are shown in **Figures 1A, B**, respectively.

The movement of the end of the bud picker can be adjusted by *XYZ* and *θ*. The manipulators move the end of the bud picker to a small picking area in the *XYZ* spatial coordinate and adjust its height according to the depth of the tea ridges. In the picking area, the surfaces of the tea ridges can be set to be flat under the same height. Meanwhile, the consumed time and the lost energy during the picking process are mainly caused by the movement of the end of the bud picker in the *XOY* plane. For convenience, we only consider the picking path planned in the *XOY* plane.

**Figure 2:**
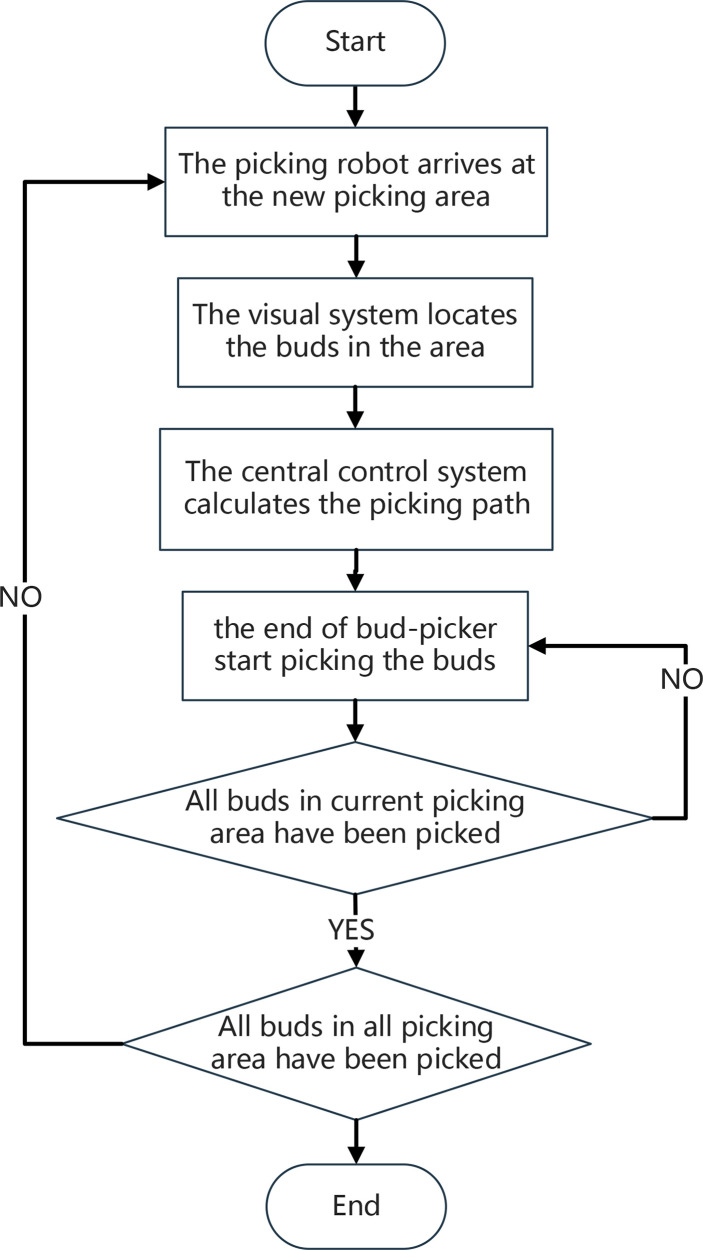
The working flowchart of the picking robot.

When the end of the bud picker moves to a picking area, the foothold parameters of a picking box are fine-tuned to complete a picking. After all buds in the picking area have been picked and collected, the manipulator drives the end of the picker to the next picking area to repeat the action. The picking scheme is divided into three steps: 1) Solving the optimal coverage picking box set, i.e., covering all of the buds in a picking area with the least number of picking box sets and obtaining the foothold parameters of each picking box; 2) Fine-tuning the foothold parameters of each picking box in the optimal coverage picking box set, so that the buds can be concentrated in the picking box as much as possible; 3) According to the foothold parameters of each picking box in the optimal coverage picking box, the end of the bud picker is planned for the shortest picking path. **Figure 2** shows the specific picking process.

### Mathematical model of the end of the bud picker

#### Picking area

**Figure 3:**
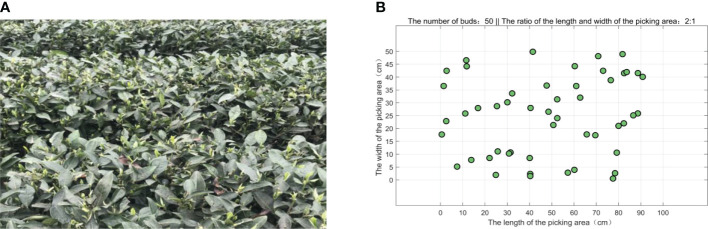
The distribution of the tea buds in a picking area. **(A)** The actual distribution of the buds in the field. **(B)** The simulated distribution of 50 buds in a picking area.

In the tea garden of Mingshan district, Ya’an city, Sichuan province, the actual typical distribution of the buds on the tea ridge is shown in **Figure 3A**. In a small picking area, 50 buds are randomly generated according to the natural distribution and numbered in sequence, as shown in **Figure 3B**. In the actual experiment, multiple areas are selected for testing, and **Figure 3** is just one of them. The length and the width of the picking area are set as 100 and 50 cm, respectively, and the ratio of the length to the width is 2:1, which basically conforms to the natural situation. Furthermore, for convenience, we have simplified the parameters as shown in **Table 1**.

**Table 1:** Notations.

Variable	Meaning
*Tea_i_*	The *i*th bud
*x_i_*	*x* coordinate of the *i*th bud
*y_i_*	*y* coordinate of the *i*th bud
*N*	The number of all buds in a peaking area
*D*	The diagonal length of a picking box
*p_0_*	The single-point picking rate
*box_i_*	The *i*th picking box
*X_i_*	*X* coordinate of the center point of the *i*th picking box
*Y_i_*	*Y* coordinate of the center point of the *i*th picking box
*θ_i_*	The included angle between the long side of the *i*th picking box and X-axis
*L*	The length of a picking box
*W*	The width of a picking box
*K*	The length-to-width ratio of a picking box
*BOX_MWC_*	The widest coverage picking box set
*BOX_BEST_*	The optimal coverage picking box set
*NUM_Tea-in_* (*box_i_*)	The number of the buds covered by *box_i_*
*NUM(A)*	The number of the elements in the *A*th set
*TC_ij_*	The bud pair formed by the *i*th bud and the *j*th bud
*d_ij_*	The length of *TC_ij_*
*α_ij_*	The included angle between *TC_ij_* and X*-*axis
*BX_i_*	The set of numbered buds that can form a bud pair with the *i*th bud (the distances from these buds to the *i*th bud are less than the diagonal of a picking box)
*φ_i_*	The angle between the left diagonal of *box_i_* and X-axis
*DDR_ij_*	The range of *φ_i_* when *box_i_* covers *TC_ij_*
*MIN_ij_*	The lower limit of *DDR_ij_*
*MAX_ij_*	The upper limit of *DDR_ij_*
*DDR_subset_*	The range of *φ_i_* while *box_i_* covers as many buds (including *Tea_i_*) as possible
*X_bxm_*	*X* coordinate of the center of the minimum bounding box
*Y_bxm_*	*Y* coordinate of the center of the minimum bounding box
*θ_bxm_*	The included angle between the long side of the minimum bounding box and X-axis
*a*	The length of the minimum bounding box
*b*	The width of the minimum bounding box
*N_picked_*	The number of picked buds in a picking area
*N_box_*	The number of picking used to pick all buds in a picking area

### Picking box

**Figure 4:**
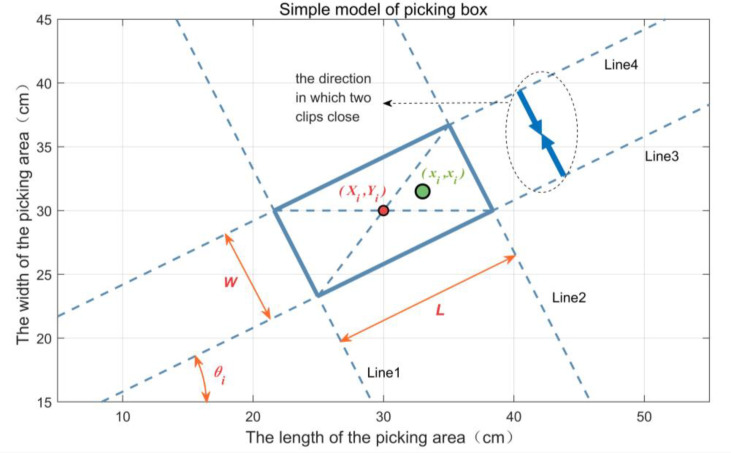
The picking box of the end of a bud picker.

In order to minimize the specific structure of the end of the bud picker and focus on the impact of the end of the picking effect, we abstracted it mathematically and proposed a simplified model of the end of the bud picker with the structure in **Figure 4**, which was named the picking box. *L* is the length of the clamping finger, and *W* is the maximum opening of two clamping fingers. (*X_i_*,*Y_i_*) are the center coordinates of the *i*th picking box. *θ_i_* is the deflection angle in the coordinate of the picking box (i.e., the included angle between the long side of the *i*th picking box and X-axis), denoted by the *i*th picking box as *box_i_* (*X_i_*,*Y_i_*,*θ_i_*,*L*,*W*) and briefly as *box_i_*. *p*_0_ is the single-point picking rate, and the parameters such as (*X_i_*,*Y_i_*,*θ_i_*) are the foothold parameters of *box_i_*. (*x_i_*,*y_i_*) are the coordinates of the *i*th bud in *box_i_*, which are within the range of Line1~Line4 in **Figure 4**.

In **Figure 4**, the mathematical model of a picking box is as follows:


(1) $$\{\begin{array}{l} \begin{array}{c} \left|y_{i}-\tan\theta_{i}(x_{i}-X_{i})-Y_{i}\right|\leq \frac{W}{2\cos\theta_{i}}\\ \left|y_{i}+\frac{1}{\tan\theta_{i}}(x_{i}-X_{i})-Y_{i}\right|\leq \frac{L}{2\sin\theta_{i}} \end{array}\;\},\theta_{i}\in -\text{π},\text{π}],\theta_{i}\neq \frac{k\text{π}}{2},k\in \{-2,-1,0,1,2\}\\ \begin{array}{l} |x_{i}-X_{i}|\leq \frac{L}{2}\\ |y_{i}-Y_{i}|\leq \frac{W}{2} \end{array}\},\theta_{i}=\frac{k\text{π}}{2},k\in \{-2,0,2\}\\ \begin{array}{l} |x_{i}-X_{i}|\leq \frac{W}{2}\\ |y_{i}-Y_{i}|\leq \frac{L}{2} \end{array}\},\theta_{i}=\frac{k\text{π}}{2},k\in \{-1,0,1\} \end{array}$$

where *i* = 1, …, *N* (*N* is the number of all buds in a picking area).

Equation 1 means that the *i*th bud can be picked by *box_i_* (*X_i_*,*Y_i_*,*θ_i_*,*L*,*W*) with the probability of *p*_0_, and the probability of each bud being picked is equal to and independent of each other:


(2) $$Tea(x_{i},y_{i})\overset{p_{0}}{\to }box(X_{i},Y_{i},\theta_{i},L,W)$$

Alternatively, briefly as:


(3) $$Tea_{i}\overset{p_{0}}{\to }box_{i}$$

### The optimal coverage picking box set *BOX_BEST_*

The combined multipoint picking scheme is as follows:

1) Traverse the *i*th bud and use it as a reference point to solve *box_i_*. *box_i_* can certainly cover the *i*th bud and cover as many other buds as possible, thus the widest coverage picking box set *BOX_MWC_* can be constructed as:


(4) $$BOX_{MWC}=\{box_{i}|i=1,2,3\ldots N\}$$

where *BOX_MWC_* means that each bud can be covered by at least one picking box ^]^ (Wei and Han, 2016), and the picking box can cover as many other buds as possible. The picking boxes in *BOX_MWC_* should meet the following conditions:


(5) $$\{\begin{array}{l} \underset{box_{i}}{\max}\;NUM_{Tea-in}(box_{i})\\ s.t.\;Tea_{i}\overset{p_{0}}{\to }box_{i} \end{array}$$

where *NUM_Tea – in_* (*box_i_*) is the number of the buds covered by *box_i_*.

2) Use the greedy algorithm to select the optimal coverage picking box set *BOX_BEST_* from *BOX_MWC_* to minimize the number of picking boxes, and *BOX_BEST_* can cover all buds in a picking area:


(6) $$BOX_{BEST}\subset BOX_{MXC}$$

This means that *BOX_BEST_* is a subset of *BOX_MWC_*.

The constraint condition is described as follows:


(7) $$\{\begin{array}{l} \underset{CUT_{BEST}}{\min}NUM(BOX_{BEST})\;\\ s.t.\;NUM_{Tea-in}(BOX_{BEST})=N\\ \;L,W=Constant \end{array}$$

where *NUM_Tea – in_* (*BOX_BEST_*) is the number of the buds covered by *BOX_BEST_*.

### Solution of *BOX_MWC_*

#### Constructing the bud pairs

A bud pair refers to two buds that can be picked together at one time by the end of the bud picker, and it is a reference point for selecting the foothold parameters of each picking box. Then, the *BOX_MWC_* is solved. To obtain *box_i_* of the *i*th bud, two-point picking needs to be solved first. Given that the diagonal length of *box_i_* is *D* when the *i*th bud and the *j*th bud can be picked together, the constraint condition is as follows:


(8) $$d_{ij}\leq D$$

Then, a directed line segment is constructed, and it is recorded as a bud pair *TC_ij_*:


(9) $$TC_{ij}=(d_{ij},\alpha_{ij})$$

where


(10) $$d_{ij}=\sqrt{{\left(x_{i}-x_{j}\right)}^{2}+{\left(y_{i}-y_{j}\right)}^{2}}$$

(11) $$\alpha_{ij}=\{\begin{array}{l} \arctan\left(\frac{y_{j}-y_{i}}{x_{j}-x_{i}}\right),x_{i}\neq x_{j}\\ \frac{\text{π}}{2},x_{i}=x_{j}\;and\;y_{i}\geq y_{j}\\ -\frac{\text{π}}{2},x_{i}=x_{j}\;and\;y_{i}<y_{j} \end{array}$$

where *α*_*ij*_∈(−*π*,*π*) is the angle between *TC_ij_* and X-axis.

All of the buds are gone through to form bud pairs and are numbered. The *i*th bud is taken as an example, its alternative bud set is named *BX_i_*, in which each bud can be formed into a bud pair with the *i*th bud. The buds in *BX_i_* can be covered by *box_i_*:


(12) $$BX_{i}=\{j|d_{ij}\leq D\}$$

**Figure 5:**
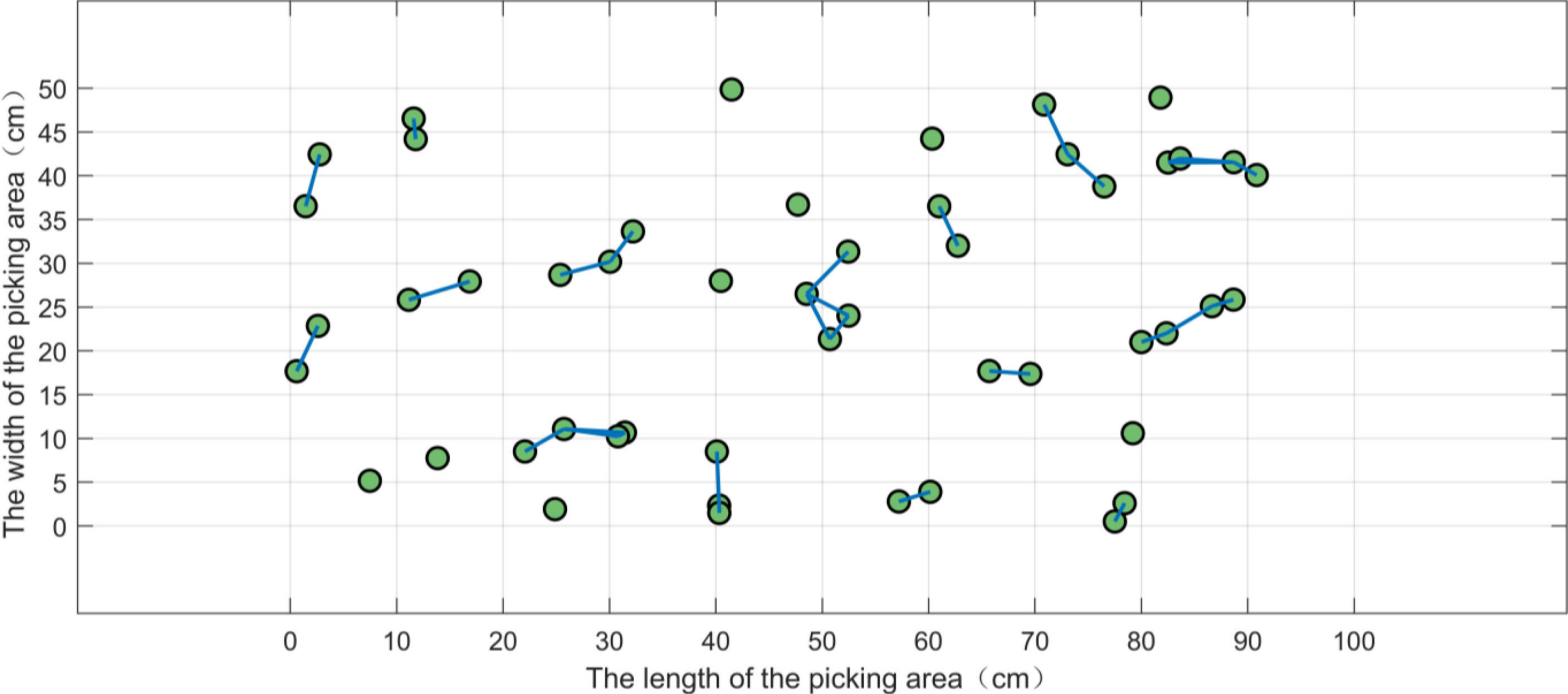
The bud pairs.

When *D* is set as $2\sqrt{10}$ cm with *L* = 6 cm and *W* = 2 cm, the bud pairs can be constructed from **Figure 1** as shown in **Figure 5**. From **Figure 5**, we know that there are 25 pairs of buds in a picking area.

### Solution of foothold parameters of *box_i_*

**Figure 6:**
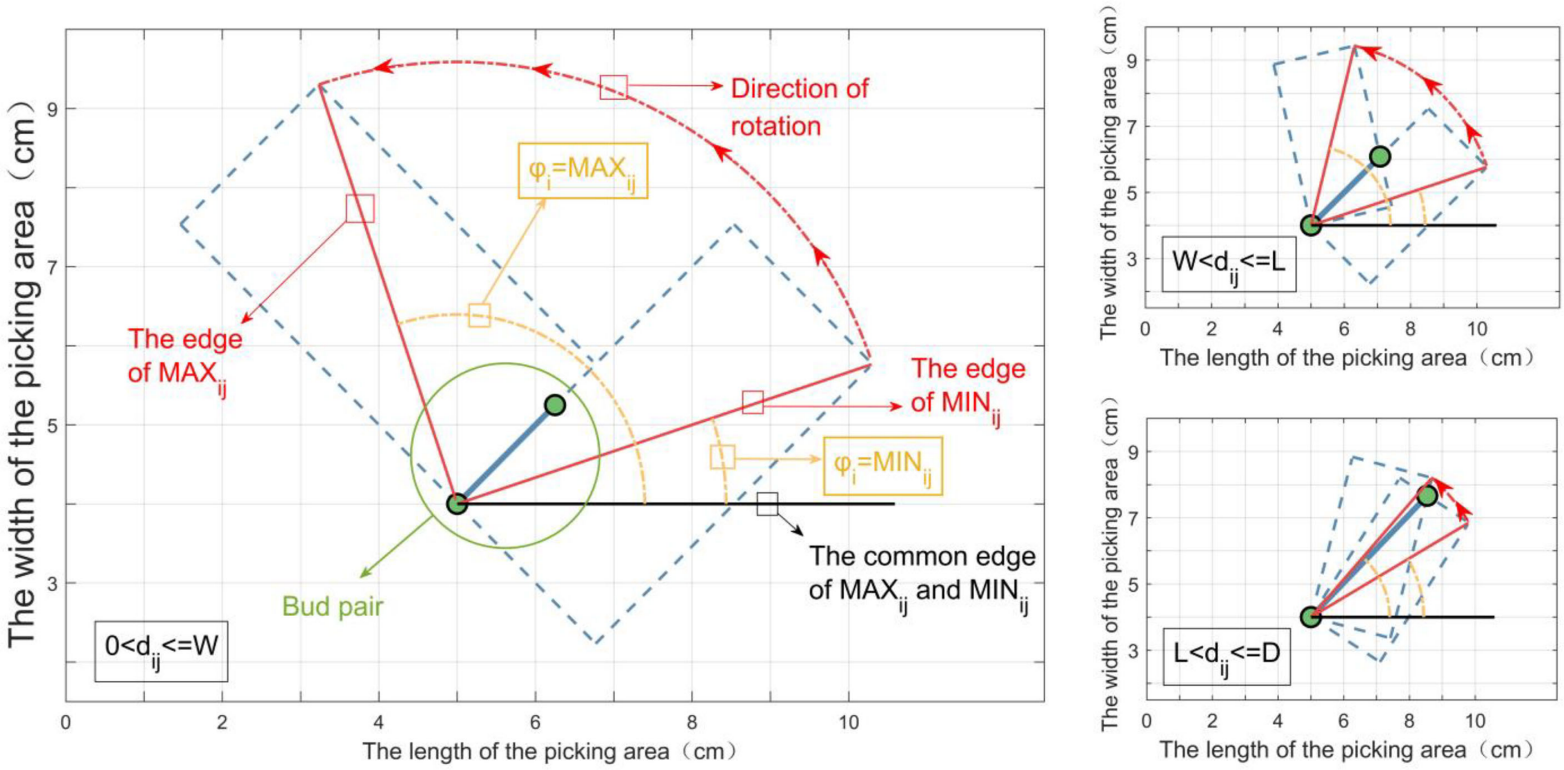
The minimum/maximum angles formed by the rotating action of *box_i._*.

According to Equation 1, when the length *L* and the width *W* of *box_i_* have been determined, its foothold parameters (*X_i_*,*Y_i_*,*θ_i_*) have not yet been confirmed. If the enumeration judgment is traversed with a certain accuracy of (Δ*X*, Δ*Y*, Δ*θ*), the time complexity of algorithm is up to *O* (*n*^3^). However, if there are too many buds, the high-speed decision requirement cannot be met. To solve this problem, the starting point of *TC_ij_* is taken as the circle center, which coincides with the lower left vertex of *box_i_*. The angle between the left diagonal of *box_i_* and X-axis is denoted by *φ_i_* when *TC_ij_* is covered by *box_i_*, and the maximum value of *φ_i_* is denoted as *MAX_ij_*. Similarly, the minimum value of *φ_i_* is denoted as *MIN_ij_*. Under different values of *d_ij_*, *MIN_ij_* and *MAX_ij_*, formed by the rotating action of *box_i_*, are shown in **Figure 6**.

According to geometric knowledge, we have the following:


(13) $$\begin{array}{l} MIN_{ij}=\{\begin{array}{l} \alpha_{ij}-\arctan\left(\frac{W}{L}\right)\;d_{ij}\leq L\\ \alpha_{ij}+\arccos\left(\frac{L}{d_{ij}}\right)-\arctan\left(\frac{W}{L}\right)d_{ij}>L \end{array}\\ MAX_{ij}=\{\begin{array}{l} \alpha_{ij}+\arctan\left(\frac{W}{L}\right)\;d_{ij}\leq W\\ \alpha_{ij}-\arccos\left(\frac{L}{d_{ij}}\right)+\arctan\left(\frac{W}{L}\right)d_{ij}>W \end{array} \end{array}$$

According to Equation 13, the rotation range of the left diagonal of *box_i_* can be obtained, and it is denoted as follows:


(14) $$DRR_{ij}=[MIN_{ij},MAX_{ij}]$$

For two bud pairs such as *TC_ij1_* and *TC_ij2_* with the same starting point (where the *j_1_th* bud and the *j_2_th* bud*∈BX_i_*), they can be picked together by the *box_i_* when *DRR_ij1_* and *DRR_ij2_* have an intersection. If a subset A of the set *{DRR_ij_|j∈BX_i_}* can be found with the most elements and all elements in A have a common non-empty intersection *DRR_subset_*, the *box_i_* can cover as many buds as possible including the reference bud *Tea_i_*. It is transformed into the following optimization problem:


(15) $$\{\begin{array}{l} \underset{DRR_{subset}}{\max}\;NUM(A)\\ s.t.\;\;A\subset \{DRR_{ij}|j\in BX_{i}\}\\ \;\overset{NUM(A)}{\underset{k=1}{\cap }}A(k)=DRR_{subset}\\ \;DRR_{subset}\neq \emptyset \end{array}$$

where *A*(*k*) is the *k*th element of *A*.

By randomly selecting *φ_i_*, the foothold parameters (*X_i_*,*Y_i_*,*θ_i_*) of *box_i_* corresponding to *Tea_i_* can be obtained, which are calculated as follows:


(16) $$\{\begin{array}{l} X_{i}=\frac{1}{2}D\cdot \cos(\theta_{i})+x_{i}\\ Y_{i}=\frac{1}{2}D\cdot sin(\theta_{i})+y_{i}\\ \theta_{i}=\varphi_{i}+\arctan\left(\frac{W}{L}\right) \end{array}$$

Furthermore, *DRR_subset_* can be solved by the interval scanning method below.

**Figure 7:**
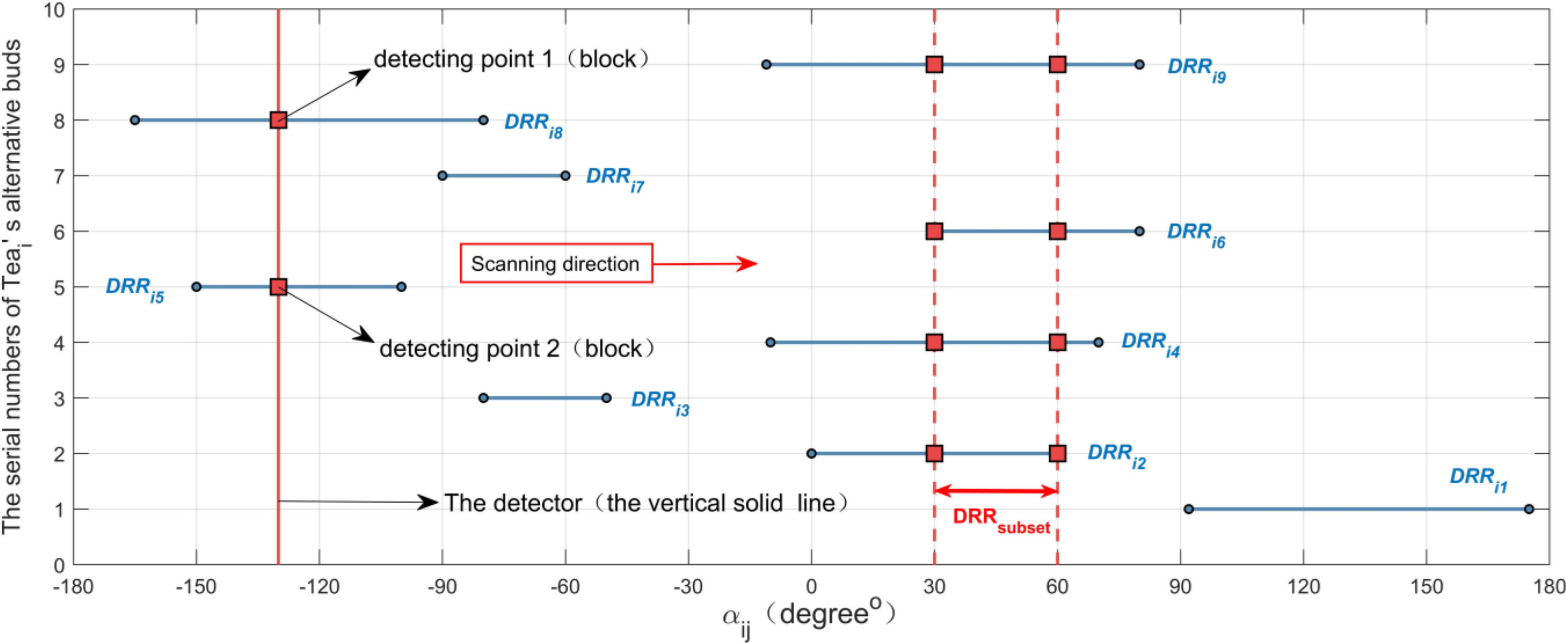
Schematic diagram of the interval scanning method.

As shown in **Figure 7**, suppose that there is a detector (i.e., the red vertical solid line in **Figure 7**) that scans counterclockwise with a certain step length. When it intersects the *DRR_ij_* (i.e., the blue horizontal solid line in **Figure 7**, *j* = 1,2,…,10), it is marked as a detection point (i.e., the red square in **Figure 7**) at the intersection. Each time the detector moves, the detection point is counted once. When the number of detection points increases, the lower limit of *DRR_subset_* is updated to the α-axis coordinate of the position of the detector; when the number of detection points does not change, the upper limit of *DRR_subset_* is updated to the α-axis coordinate of the position of the detector; if the number becomes smaller, it will not be updated. After detector scanning for a circle, *DRR_subset_* is recorded as [30^0^, 60^0^] in **Figure 6**. Foothold parameters (*X_i_*,*Y_i_*,*θ_i_*) of *box_i_* can be calculated for *Tea_i_*, while *φ_i_* takes the middle value of *DRR_subset_*. Using the same way to traverse each bud, the widest coverage picking box set *BOX_MWC_* can be obtained finally.

**Figure 8:**
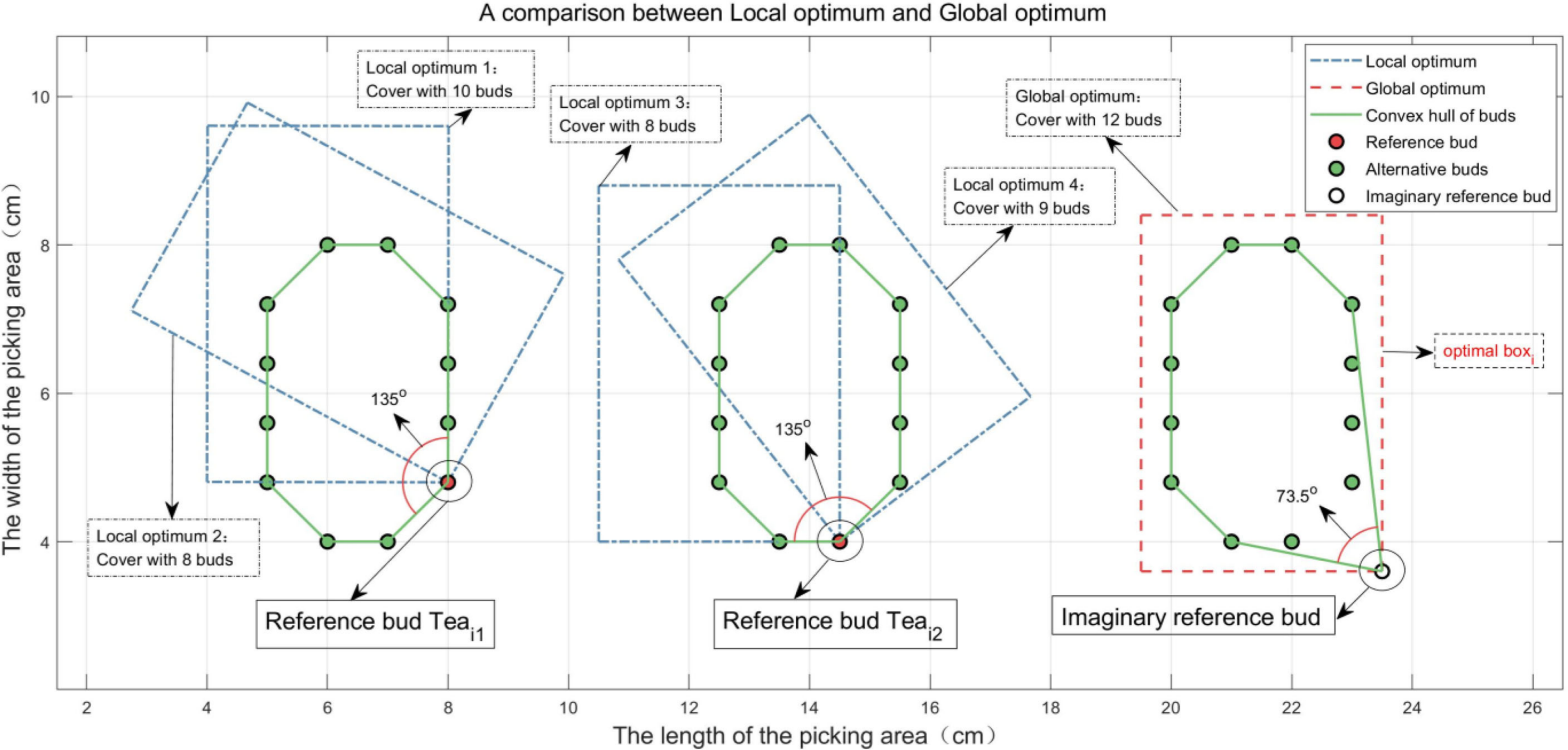
Comparison of local optimum and global optimum.

Foothold parameters (*X_i_*,*Y_i_*,*θ_i_*) of *box_i_* calculated by the above method are not necessarily a globally optimal solution. As shown in **Figure 8**, the inner angles of the convex hull of the point set formed by the reference bud (i.e., *Tea*_*i*_1__ or *Tea*_*i*_2__ shown in **Figure 8**) and its alternative buds (i.e., {*Tea*_*j*_1__|*j*_1_∈*BX*_*i*_1__} or {*Tea*_*j*_2__|*j*_2_∈*BX*_*i*_2__} ) that can be picked together are greater than 90°, which will lead to local optimums. No matter how *box_i_* rotates, it cannot cover all buds. If the inner angle of the convex hull of the point set formed by the imaginary reference bud in **Figure 8** and its alternative buds is not more than 90^0^, the ideal *box_i_* can cover all buds, which means that foothold parameters of *box_i_* can be calculated preferably (Cheein and Guivant, 2014; Gao et al., 2015).

As shown in **Figure 8**, although the probability that the inner angles are greater than 90^0^ is extremely small in fact, the opposite situation still happens (i.e., *Tea*_*i*_1__ or *Tea*_*i*_2__ shown in **Figure 8**) occasionally, in which the buds can be covered by two picking boxes. This situation only uses one more picking box than ideal. In **Figure 8**, the third and fourth local optimums of *box_i_* are superimposed to completely cover 12 buds to be picked. Therefore, the above method is sufficient to meet the actual picking demand.

### Solution of *BOX_BEST_*

*BOX_BEST_* means to cover all buds in a picking area with the least number of picking boxes. According to Equation 6, *BOX_BEST_* is a subset of *BOX_MWC_*. To traverse each *box_i_* in *BOX_MWC_*, use Equation 1 to find the buds covered by *box_i_*, record their numbers, and finally establish a bud set *HC_i_* as follows:


(17) $$HC_{i}=\{j|Tea_{j}\overset{p_{0}}{\to }box_{i}\},i=1,\cdots ,N$$

**Figure 9:**
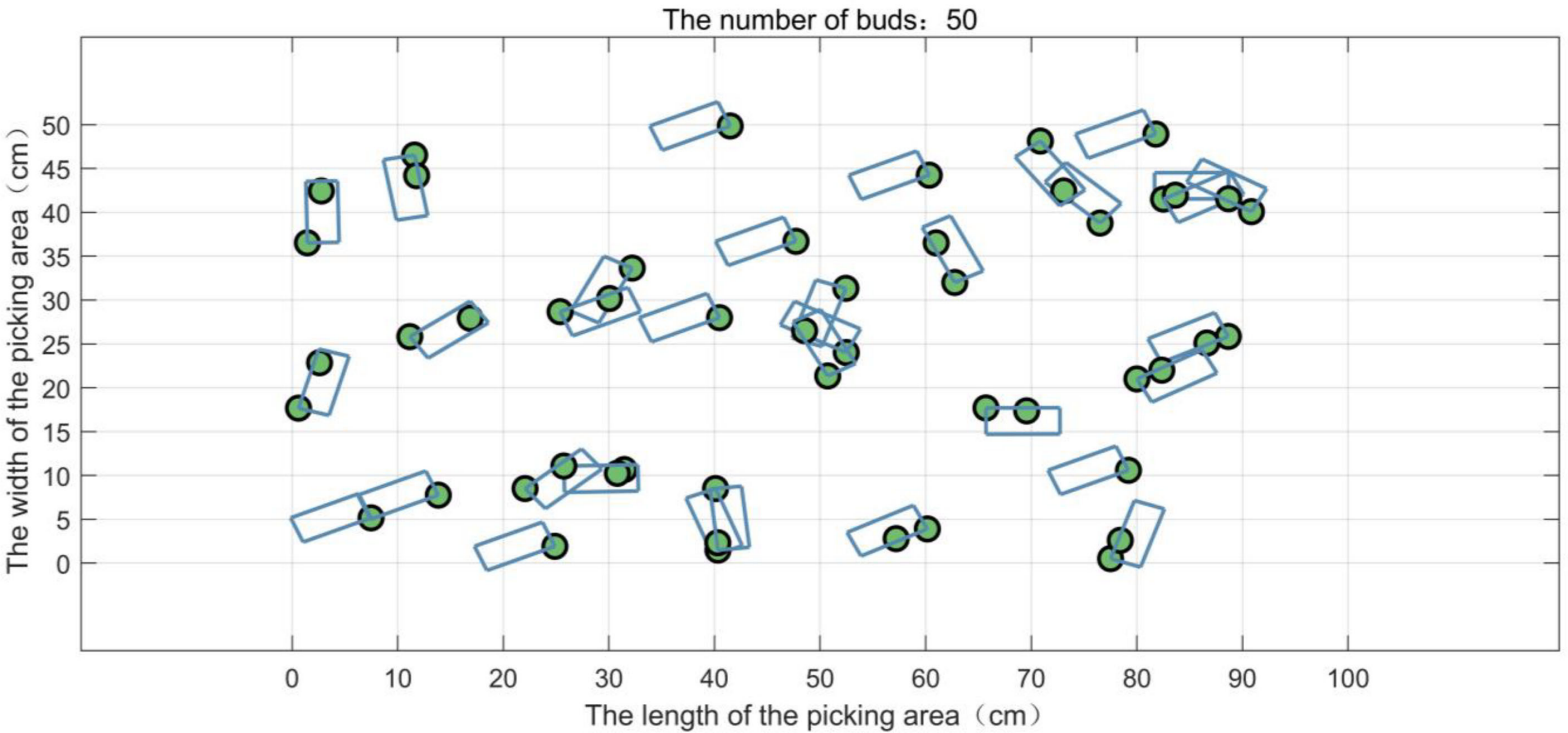
The picking boxes in *BOX_BEST_* (before fine-tuning).

The greedy algorithm is used to obtainCUTBESTand the steps are as follows (Tong et al., 2017; Zhao et al., 2017; Zhang et al., 2018). Firstly, find the set with the most elements in *HC_i_* (*i*=1,2,…*N*) and record its subscript *i_most_*. Secondly, remove all elements of the set *HC*_*i*_*most*__ and remove the elements of *HC_i_* (*i* ≠ *i_most_*) that are also of *HC*_*i*_*most*__ . Thirdly, put *box*_*i*_*most*__ into *BOX_BEST_*. Repeat the above steps until the number of elements in all sets (*HC*_1_,*HC*_2_,…,*HC_N_*) is zero, and then *BOX_BEST_* can be obtained finally. The picking boxes in *BOX_BEST_* are shown in **Figure 9**.

### Fine-tuning the foothold parameters of picking boxes in BOX_BEST_

*BOX_BEST_* essentially classifies all buds, and the buds that can be covered by the same picking box are classified into one category. **Figure 9** shows that many buds are on the edges of the picking boxes, so it is necessary to fine-tune the foothold parameters of picking boxes in *BOX_BEST_* to make the most buds concentrated in the middle of the picking boxes as much as possible and thus ensure the integrity of buds after being picked together.

For the bud set covered by a picking box in *BOX_BEST_*, use the Graham algorithm to solve the convex hull of the bud set (Song et al., 2012; Zhang et al., 2019) and then apply the convex hull to calculate the minimum bounding box of the bud set (Park and Lim, 2014; Tang et al., 2018). The central coordinate of the minimum bounding box is (*X_bxm_*,*Y_bxm_*), and the angle between the long side of the minimum bounding box and X-axis is *θ_bxm_*. The length and width of the minimum bounding box are *a* and *b*, respectively. Then, (*X_bxm_*,*Y_bxm_*,*θ_bxm_*) are the fine-tuned foothold parameters of each picking box in*BOX_BEST_*, and *a b* meet the following constrained conditions:


(18) $$\{\begin{array}{c} \underset{a,b}{\min}ab\\ \begin{array}{ccc} s.t. & a\leq L\;, & b\leq W \end{array} \end{array}$$

According to Cheng et al. (2008), if a point set has the minimum bounding box, an edge of its minimum bounding box must coincide with an edge of its convex hull. By this, traverse each edge *l_k_* (*k* = 1,2,…,*n*) of the convex hull, then take *l_k_* as the reference edge, and make the circumscribed rectangle of the point set, so that one edge of the rectangle coincides with *l_k_*, and the parameters of the rectangle can be obtained by geometric knowledge. A set of parameters satisfying the condition of Equation 18 is the parameter (*X_bxm_*,*Y_bxm_*,*θ_bxm_*,*a*,*b*) of the minimum bounding box (Yan et al., 2013).

**Figure 10:**
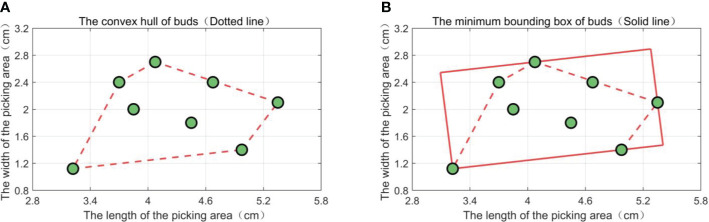
Convex hull of the bud set and its minimum bounding box. **(A)** A diagram of the convex hull of a point set. **(B)** A diagram of the minimum bounding box of a point set.

Taking a randomly distributed bud set of eight buds as an example, the convex hull of the point set obtained by the Graham algorithm is shown in **Figure 10A**, and the minimum bounding box of the bud set is shown in **Figure 10B**.

**Figure 11:**
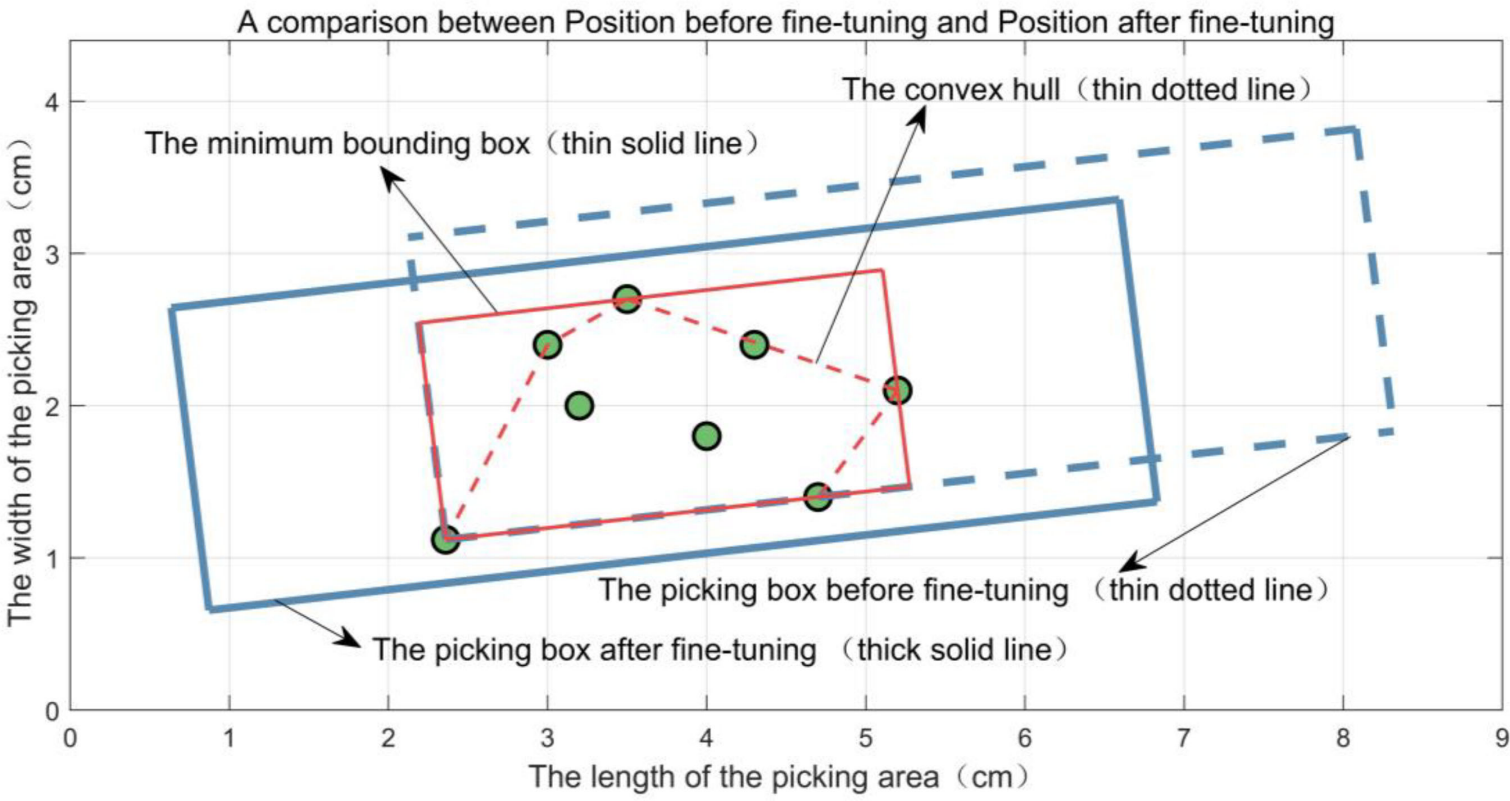
Position comparison of a picking box before and after fine-tuning its foothold parameters.

The parameters (*X_bxm_*,*Y_bxm_*,*θ_bxm_*) of the minimum bounding box are obtained as the fine-tuned foothold parameters of the corresponding picking box in *BOX_BEST_*. The position comparison before and after the fine-tuning of the picking box’s footing point parameters is shown in **Figure 11**. From **Figure 11**, it can be seen that the buds are concentrated in the middle of the fine-tuned picking box.

## Results and discussion

### Solving the shortest path

The optimal foothold parameters of a picking box (i.e., the end of the bud picker) have been obtained in the foregoing. In order to further improve the picking efficiency, the ant colony algorithm is applied to plan the optimal picking path of the end of the bud picker (Jiang et al., 2015; Wang et al., 2020; Lin et al., 2020). The information track persistence parameter is adaptively changed (Wen et al., 2017; Deng et al., 2018; Wang L. et al., 2018), and the other parameters of the ant colony algorithm are screened out after many experiments (see **Table 2** for details).

**Table 2:** Parameters of the ant colony algorithm.

Number of ants	Importance of pheromone	Importance of heuristic factor	Information volatility coefficient	Pheromone intensity increasing coefficient
12	0.95	0.90	0.30	100

**Figure 12:**
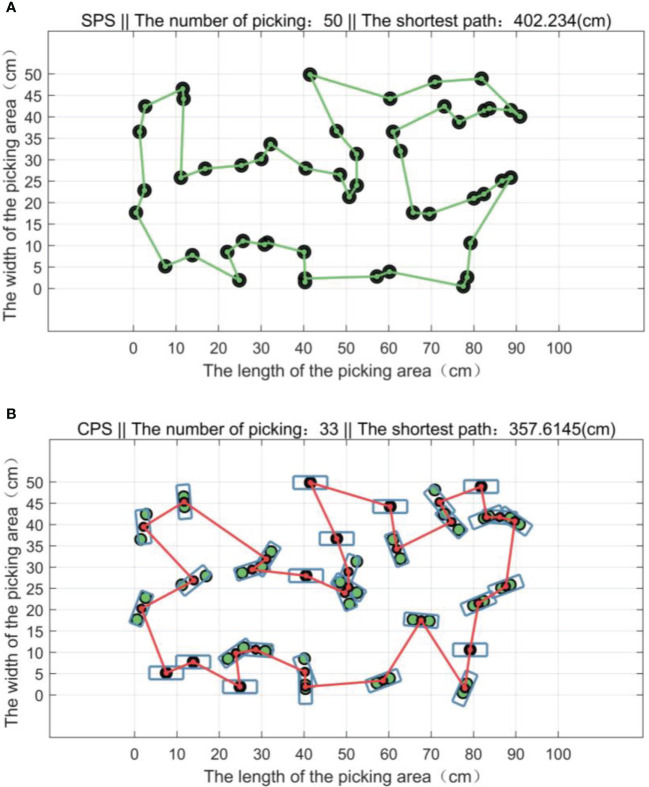
The shortest paths of two picking schemes. **(A)** The shortest paths of the single-point picking scheme (SPS). **(B)** The shortest paths of the combined multipoint picking scheme (CPS).

When the end of the bud picker moves on the *XOY* plane, its rotation angle *θ* can be adjusted synchronously. When planning the picking path with the goal of shortening the consumed time, the adjusting time of *θ* can be ignored, and it is regarded as planning the picking path of the manipulator in the XOY plane. Set the length *L* = 6 cm and the width *W* = 2 cm of a picking box for simulation. The CPU is the Intel core i5-6300HQ processor with the memory capacity of 4 GB. The results of the single-point picking scheme (abbreviated as SPS) and the combined multipoint picking scheme (abbreviated as CPS) are shown in **Figure 12**.

**Figure 13:**
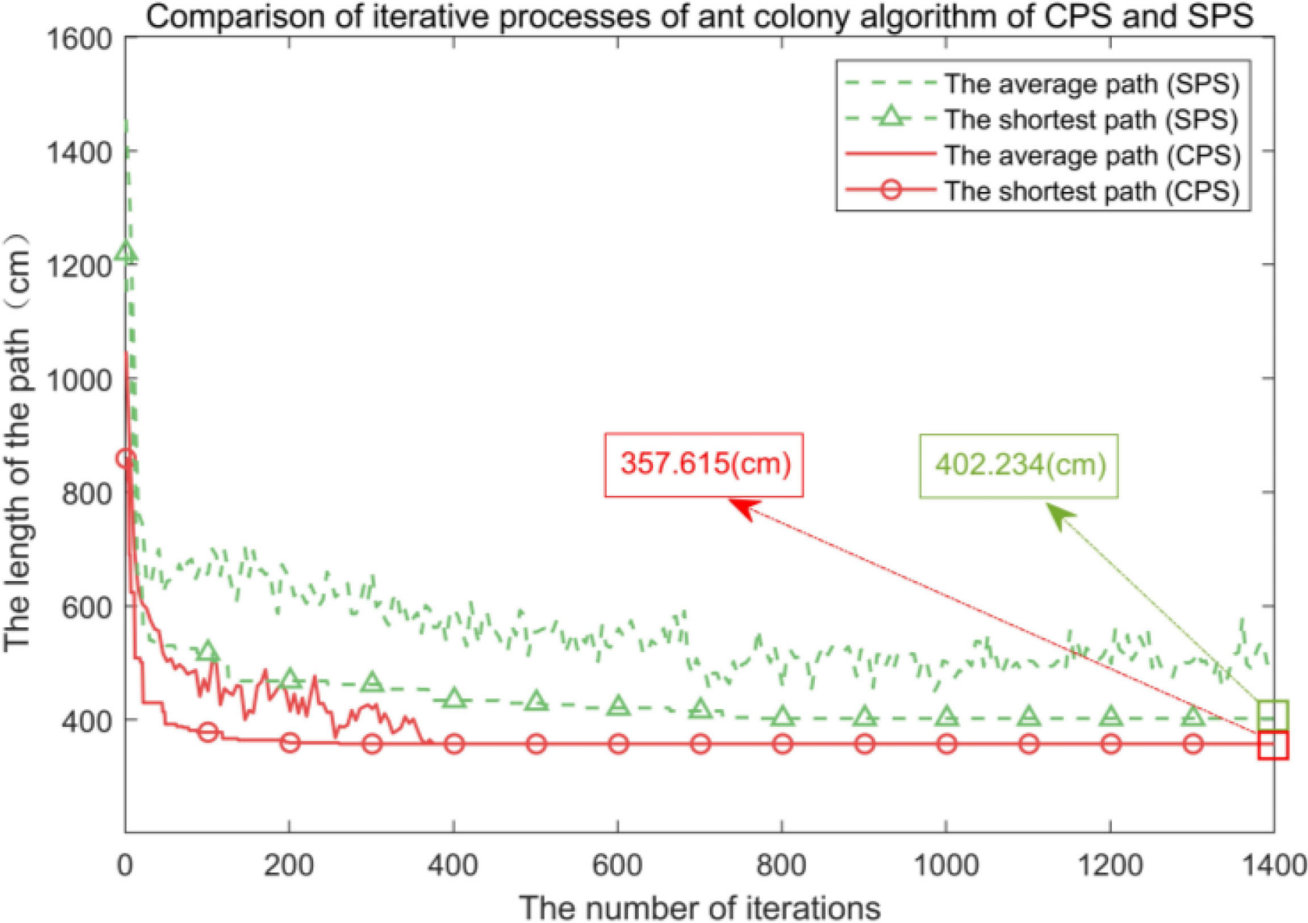
Comparison of the average shortest picking path and the shortest picking path of the two picking schemes.

In a picking area, 50 buds are randomly distributed 100 times, and the experiment is repeated. Experimental results of the two picking schemes are shown in **Figure 13**, and the statistical results are listed in **Table 3**.

**Table 3:** Comparison of 100 experimental statistical results of the two picking schemes.

Scheme	Average consumed time (s)	Standard deviation of consumed time (s)	Average number of picking	Standard deviation of number of picking	The average shortest picking path (cm)	Standard deviation of the shortest picking path (cm)
SPS	28.910	9.040	50	0	426.520	20.401
CPS	14.190	5.038	34	2.00	379.260	22.262

SPS, single-point picking scheme; CPS, combined multipoint picking scheme.

From **Figures 12**, **13** and the data in **Table 3**, it can be seen that all buds are in the middle of the picking boxes and 1–2 buds can be picked successfully at a time. Compared with the SPS, the average number of picking of the CPS is reduced by 31.44%, the shortest picking path is decreased by 11.10%, and the average consumed time is reduced by 50.92%, so its picking performance is better than that of the SPS.

The data in **Table 3** were tested by the *Z* hypothesis test to analyze the significant differences between the two schemes in terms of the consumed time, the number of picking, and the shortest picking path. The *Z*-test was calculated as follows:


(19) $$Z=\frac{\left|\bar{X_{SPS}}-\bar{X_{CPS}}\right|}{\sqrt{\frac{S_{SPS}^{2}}{n_{ex}}+\frac{S_{CPS}^{2}}{n_{ex}}}}$$

where $\bar{X_{SPS}}$ and $\bar{X_{CPS}}$ are the mean values of the specific data in the SPS scheme and the CPS scheme, respectively, *S_SPS_* and *S_CPS_* are the standard deviations of the specific data in the SPS scheme and the CPS scheme, respectively, and *n_ex_* is the number of experiments that is set to 100。.

According to the hypothesis test table, when *Z* is greater than 1.96, it is corresponding to the significance *P* ≤ 0.05, which indicates that there is a significant difference between the two schemes under this parameter. According to Equation 19, *Z* equals 14.22, 80.00, and 15.65 for the consumed time, the number of picking, and the shortest picking path, respectively; therefore, there are significant differences between the two schemes in the abovementioned three parameters.

### Evaluating the scheme under different parameters

#### Setting the range of parameters

If the *L*, *W*, *p*_0_, and *N* are different, the *N_picked_* and *N_box_* will be different, as follows:


(20) $$\left[N_{picked},N_{box}]=f(L,W,p_{0},N\right)$$

In order to analyze the effect of the above four parameters on the performance of the CPS scheme and optimize it, a factorial design is adopted. Different steps and ranges are set for the parameters including *L*, *W*, *p*_0_, and *N* in **Table 4**. Each group of four parameters is used in the procedure 50 times to calculate the average value. A total of 10,000 pairs of [*N_picked_*, *N_box_*] can be obtained.

**Table 4:** Parameter setting.

Parameter	Range	Step
*L*	[2–20]	2
*W*	[2–20]	2
*p*_0_	[0.1–1]	0.1
*N*	[20–200]	20

In order to obtain more accurate quantized parameters, the level number was set to 10, which will increase the difficulty of parameter analysis. Therefore, targeted analysis was made on the part of experimental results that help optimize the CPS scheme.

### Evaluation indicator

Three indicators, *Δp* (i.e., the increment of total picking rate), *Δr* (i.e., the reduction ratio of the number of picking), and *Q* (i.e., the yield per unit area), are used to evaluate the performance of the picking scheme.

1) Δ*p*. The CPS will cause a probability of repeatedly picking a bud. Δ*p* is the increment of total picking rate of the CPS compared to that of the SPS. Δ*p* is calculated as follows:


(21) $$\Delta p=\frac{N_{picked}}{N}-p_{0}$$

(22) $$N_{picked}=N-\sum_{i=1}^{N}{\left(1-p_{0}\right)}^{m_{i}}\;$$

where *m_i_* is the number that the *i*th bud is repeatedly picked.

2) Δ*r*. The less the number of picking in a picking area, the shorter the picking path. Δ*r* is the reduction ratio of the number of picking of the CPS compared to that of the SPS. Δ*r* is calculated as follows:


(23) $$\Delta r=\frac{N-N_{box}}{N}$$

3) *Q*. The smaller the area of the picking box, the higher the integrity rate of the picked buds. *p*_0_ will decrease with the increase of *S.* That is, the smallest area of a picking box can get the maximum values of Δ*p* and Δ*r*. *Q* is calculated as follows:


(24) $$Q=\frac{\Delta p\Delta r}{S}$$

where *S* is the area of a picking box.

When *Q* is the maximum value, the corresponding *L* and *W* are the optimal sizes of a picking box. It should be noted that Δ*p* = *0* when *p*_0_ = 1, the overall picking rate will no longer increase, and *Q* = 0 at this time. In order to evaluate the yield under the special case of *p*_0_ = 1, a new evaluation model is constructed as follows:


(25) $$Q_{r}=\frac{\Delta r}{S}$$

where *Q_r_* is the yield per unit area, while *p*_0_ = 1. That is, if the performance of the end of the bud picker is optimal, *Q* is usually used as the evaluating criterion. If the focus is on the improvement of picking efficiency, *Q_r_* will be used as the criterion.

The above indicators are used to select the optimal parameters including the length-to-width ratio and the area of a picking box to achieve the best picking performance under different parameter pairs (*N*, *p*_0_).

### Evaluation analysis on the parameters

#### 1) Influencing factors of Δ*p* and Δ*r*

**Figure 14:**
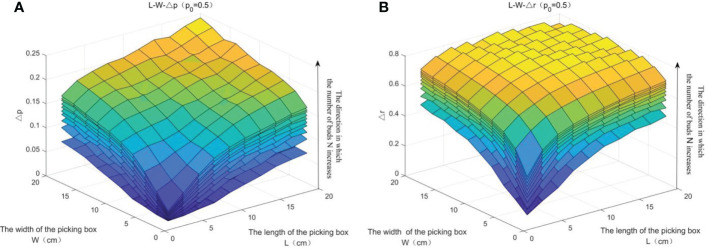
The relationship of *Δp* and Δ*r* with the parameters *W*, *L*, and *N.* **(A)** The relationship of Δp with the parameters W, L, and N. **(B)** The relationship of Δ*r* with the parameters W, L, and N.

The relationship between Δ*p* and Δ*r* with the parameters *W*, *L*, and *N* is shown in **Figure 14**. It can be seen from **Figure 14** that when the single-point picking rate *p*_0_ is constant (i.e., *p*_0_ is set as 0.5), *Δp* (*W*, *L*, *N*) and *Δr* (*W*, *L*, *N*) are both curved surfaces. When *N* is constant, *Δp* and *Δr* are both increasing with the increase of *L* and *W*. When *L* and *W* are constant, *Δp* and *Δr* are both increasing with the increase of *N*. The overall picking rate can be increased by about 25%, and the number of picking can be reduced by about 80%.

#### 2) The influence factors of *Q* and the selection of the optimal length-to-width ratio of a picking box

**Figure 15:**
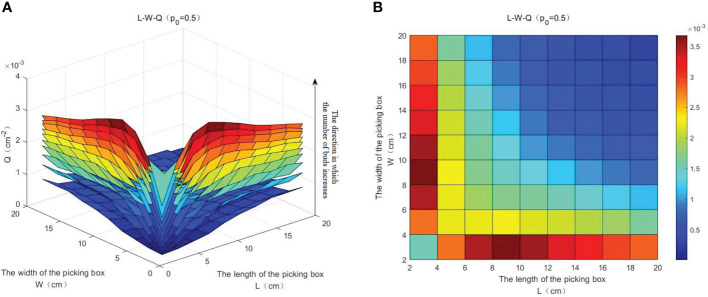
The relationship between *L*, *W*, and *Q*. **(A)** The relationship between *L*, *W*, and *Q*. **(B)** The relationship between *L*, *W*, and *Q* (top view).

The relationship between *L*, *W*, and Q is shown in **Figure 15**. It can be seen from **Figure 15** that *Q* increases with the increase of *N*, while *p*_0_ is constant (i.e., *p*_0_ = 0.5). With the increase of *L* and *W*, *Q* does not show a monotonous increasing trend, and it is significantly higher in the edge area than in the center area. The top view of **Figure 15A** is shown in **Figure 15B**, from which it can be seen that the value of *Q* in the stripe area of *W* = 2 is significantly higher than that of other areas, especially when *L* = 6 or 8, *Q* reaches the maximum value. Under different values of *N* and *p*_0_, the experimental results provide a theoretical basis for selecting the optimal length-to-width ratio of a picking box to reach the maximum value of *Q*.

**Figure 16:**
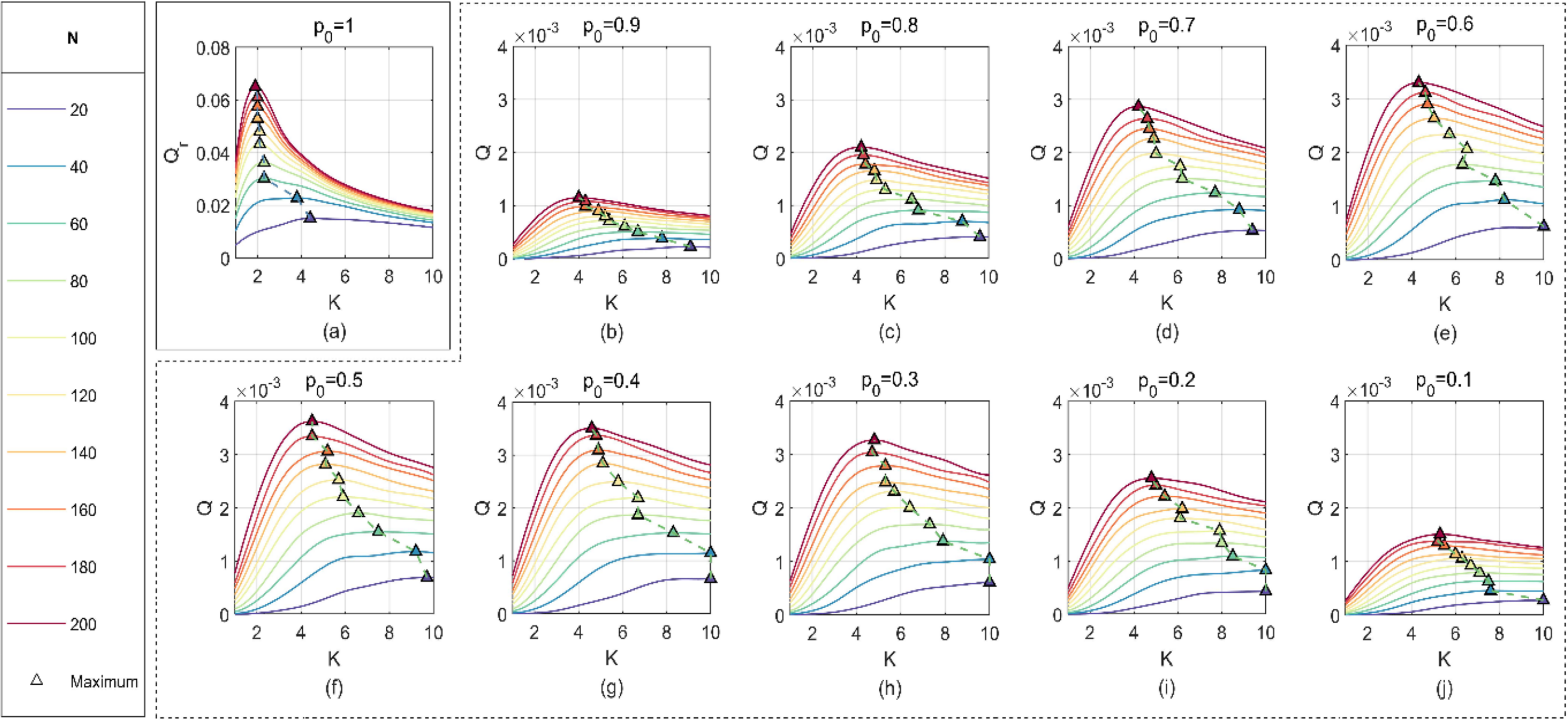
Influence of *K* on *Q_r_* and *Q* with different values of *N* and *p*_0._.

The influence of *K* on *Q* is analyzed with different values of *N* and *p*_0_. *W* is set as 2 for simulation, and the results are shown in **Figure 16**. It can be shown from **Figure 16** that *K* shows a downward trend with the increase of *N* while *Q* is the maximum, and the trend has no significant change as *p*_0_ increases from 0.1 to 0.9. When *Q* or *Q_r_* is the maximum value, the influence of *N* on *K* is shown in **Figure 16**, from which it can be seen that when *p*_0_ = 1, *Q_r_* decreases with the increase of *N* and then remains stable after *N* ≥ 60.

The least square algorithm (Wang P. et al., 2017; Zhou et al., 2019) is used to fit the points in **Figure 16**. While *p*_0_ increases from 0.1 to 0.9, the optimum value of *K* can be estimated as follows:


(26) $$K=\{\begin{array}{l} -4.765N^{0.2115}+18.92,\;take\;Q\;as\;the\;standard\\ \;133.2N^{-1.216}-1.657,\;take\;Q_{r}\;as\;the\;standard \end{array}$$

**Figure 17:**
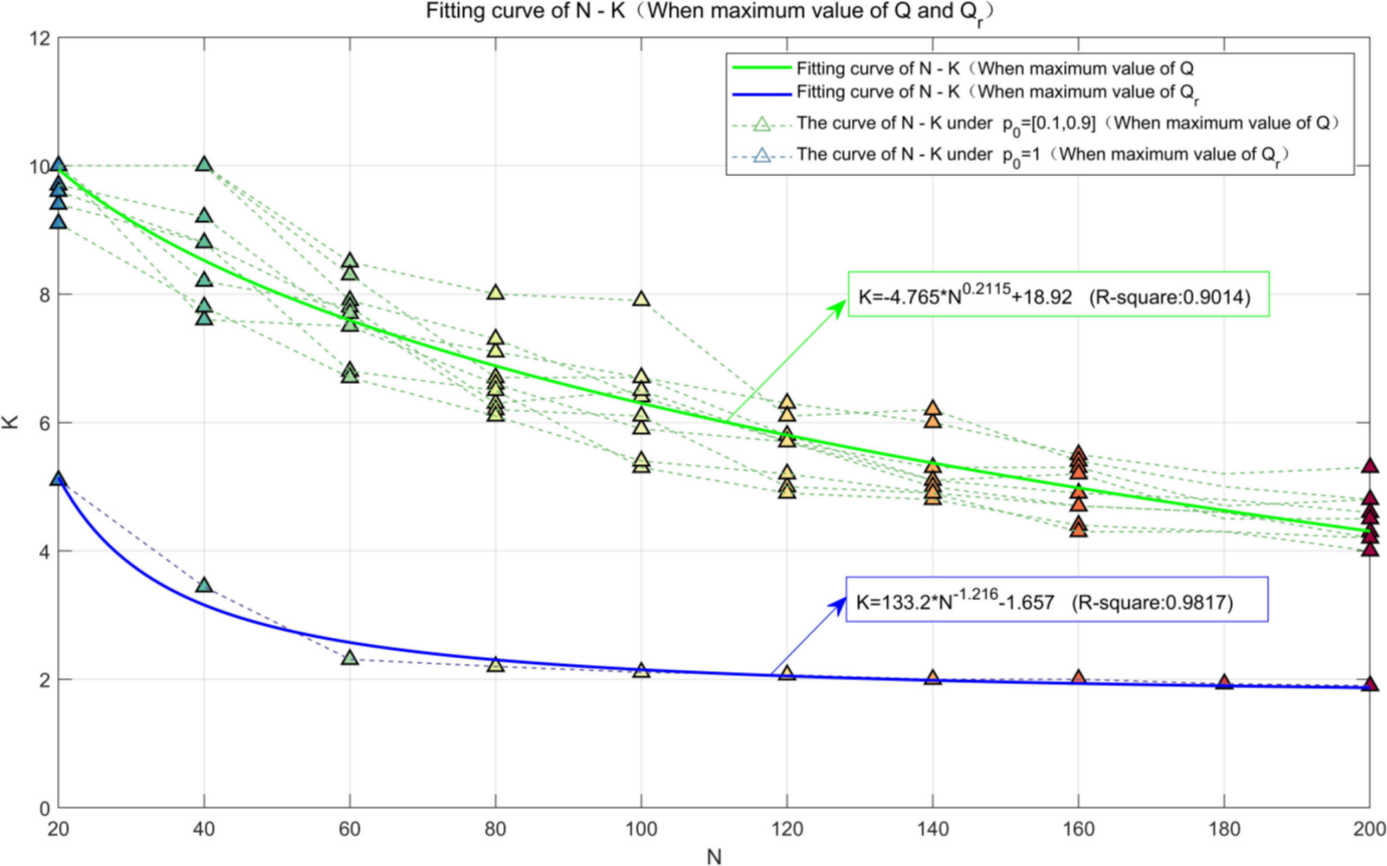
Influence of *N* on *K* when *Q* or *Q_r_* is the maximum value.

It can be seen from **Figure 17** that the optimum value of *K* is more conservatively estimated, while *Q_r_* is used as the evaluating criterion. It is worth noting that the optimum value of *K* tends to be 2 when *N* ≥ 60.

When *p*_0_ = 0.5 and *W* = 2, the performance indicators corresponding to the maximum value of *Q* are shown in **Table 5**. It can be seen from **Table 5** that as *N* increases, both Δ*p* and Δ*r* increase while the optimum value of *K* decreases gradually.

**Table 5:** Evaluation indicators (*p*_0_ = 0.5, *W* = 2, and *Q* is the maximum value).

N	The optimum value of K	Δp (%)	Δr (%)
20	8.5	6.24	44.70
40	8.0	8.00	48.00
50	7.63	8.37	49.12
60	7.30	8.81	50.43
80	6.70	9.12	50.90
100	6.20	8.70	50.94
120	5.60	9.62	53.10
140	5.00	10.16	55.56
160	4.40	10.95	56.61
180	3.90	9.82	55.02
200	3.30	11.56	56.27

#### 3) Influence of *p*_0_ and *N* on *Q*

**Figure 18:**
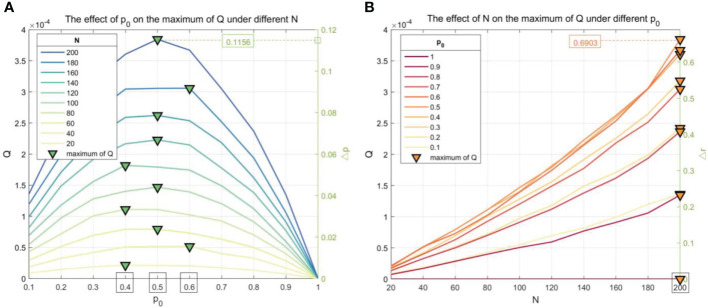
The curves of *Q* varying with *p*_0_ and *N*. **(A)** The effect of *p*_0_ on the maximum of Q under different N. **(B)** The effect of N on the maximum of Q under different *p*_0._.

The relationship between *p*_0_, *N*, and *Q* will affect the applicability of the CPS. In the actual picking process, *p*_0_ and *N* are usually constant values, and the curves of *Q* varying with *p*_0_ and *N* are shown in **Figure 18**.

When *N* = [20, 200], it can be seen from the left vertical axis in **Figure 18A** that the curve of *p*_0_-*Q* is approximately parabolic, and *Q* reaches the maximum value when *p*_0_ is 0.5, which means that the CPS is superior to the SPS obviously when *p*_0_ is closer to 0.5. The right vertical axis in **Figure 18A** shows that Δ*p* increases from 0% to 11.56% with *N* increasing from 20 to 200. It can be seen from **Figure 18B** that the curve of *N*-*Q* increases monotonously, which means that the CPS is superior to the SPS if there are more buds. The right vertical axis in **Figure 18B** shows that Δ*r* increases from 0% to 69.03% with *N* increasing from 20 to 200, which means less number of picking of the CPS.

## Conclusion

A matrix mathematical model has been established for a picking box, and its parameters were fine-tuned. The CPS was innovatively put forward for picking the buds in a picking area. Compared with the SPS, it has the characteristics of higher picking efficiency, overall picking success rate, and shorter consumed time. The results of this study are as follows:

1) Fifty buds are randomly distributed in a picking area of 100 × 50 (cm^2^), and *L* and *W* are set as 6 and 2 cm, respectively. The experimental results of 100 random distributions show that compared to the SPS, the number of picking, the shortest picking path, and the average consumed time of the CPS were greatly reduced.
2) Picking results were analyzed according to *L* and *W* of a picking box, the single-point picking rate *p*_0_, and the total number of buds *N*. The closer *p*_0_ is to 0.5, the higher the yield per unit area *Q* of CPS. When *p*_0_ = 0.5, the overall picking rate can increase from 0% to 11.56% with *N* increasing from 20 to 200.
3) Both *L* and *W* are closely related to the number of all buds in a peaking area *N*. Experimental results show that the optimal length-to-width ratio of a picking box can be calculated by *K* = -4.765*N*^0.2115^ + 18.92, so the optimal sizes of the picking box can be calculated for picking areas with different distribution densities. While the picking efficiency is more emphasized, *K* = 133.2*N*^-1.216^ – 1.657 is selected instead.

Experimental results show that the CPS greatly improved the overall picking rate and picking efficiency. In fact, to simplify the problem, the CPS abstracted the buds into some coordinate points and set a fixed single-point picking rate *p*_0_, thus resulting in a few buds that cannot be covered by the picking box and difficulty ensuring the constant value of *p*_0_ in actuality. Therefore, the follow-up study will solve the problems from three aspects: 1) Consider the sizes of buds while seeking the optimal coverage picking box set; 2) More factors that affect *p*_0_ will be analyzed further; 3) The CPS will be further demonstrated, and the optimal sizes of a picking box will be designed more accurately.

## Data availability statement

The raw data supporting the conclusions of this article will be made available by the authors, without undue reservation.

## Author contributions

LX: Conceptualization, Investigation, Writing-original draft, Writing-review and editing. XC: Conceptualization, Investigation, Writing-original draft, Writing-review and editing. YC, YX and ZK: Methodology, Analysis, Suggestion. PH, HY and ZZ: Writing-review and editing, Analysis, Suggestion. ZL and LM: Writing-review and editing, Analysis, Suggestion. JD, ZW and BL: Writing-review and editing, Analysis, Suggestion. NY, YW and YZ: Validation, Resources, Supervision, Project administration, Writing-review and editing, Funding acquisition. All authors contributed to the article and approved the submitted version.

## Funding

This study was funded by the Science and Technology Innovation Cultivation Project of Department of Science and Technology of Sichuan province (Grant No. 2021JDRC0091), the Key R & D project of Department of Science and Technology of Sichuan province (Grant No. 2020YFN0025).

## Conflict of interest

The authors declare that the research was conducted in the absence of any commercial or financial relationships that could be construed as a potential conflict of interest.

## Publisher’s note

All claims expressed in this article are solely those of the authors and do not necessarily represent those of their affiliated organizations, or those of the publisher, the editors and the reviewers. Any product that may be evaluated in this article, or claim that may be made by its manufacturer, is not guaranteed or endorsed by the publisher.
